# Confocal Identification of Immune Molecules in Skin Club Cells of Zebrafish (*Danio rerio*, Hamilton 1882) and Their Possible Role in Immunity

**DOI:** 10.3390/biology11111653

**Published:** 2022-11-11

**Authors:** Alessio Alesci, Marco Albano, Serena Savoca, Doaa M. Mokhtar, Angelo Fumia, Marialuisa Aragona, Patrizia Lo Cascio, Marwa M. Hussein, Gioele Capillo, Simona Pergolizzi, Nunziacarla Spanò, Eugenia Rita Lauriano

**Affiliations:** 1 Department of Chemical, Biological, Pharmaceutical, and Environmental Sciences, University of Messina, 98166 Messina, Italy; 2 Department of Biomedical, Dental and Morphological and Functional Imaging, University of Messina, 98125 Messina, Italy; 3 Institute for Marine Biological Resources and Biotechnology (IRBIM), National Research Council (CNR), Section of Messina, 98100 Messina, Italy; 4 Department of Anatomy and Histology, Faculty of Veterinary Medicine, Assiut University, Assiut 71526, Egypt; 5 Department of Clinical and Experimental Medicine, University of Messina, Padiglione C, A. O. U. Policlinico “G. Martino”, 98124 Messina, Italy; 6 Department of Veterinary Sciences, University of Messina, 98168 Messina, Italy; 7 Department of Cell and Tissues, Faculty of Veterinary Medicine, Assiut University, Assiut 71526, Egypt

**Keywords:** confocal microscopy, club cells, evolution, innate immunity, ostariophysi

## Abstract

**Simple Summary:**

The immune systems of fish can respond rapidly to biological, physical, and environmental stresses and defend the body against pathogens. The skin is the organ most in contact with the external environment and possesses a plethora of immune cells. Club cells, typical of the skin of Ostariophysi, are able to produce alarming substances following a predatory attack or exposure to toxins and parasites. This study aims to immunohistochemically characterize club cells in zebrafish skin for the first time with different immune molecules and adds further data on the involvement of these cells in the immune response.

**Abstract:**

The immune system of a fish has cellular and molecular defense mechanisms that are substantially retained throughout the evolution of vertebrates. The innate immune system provides biological processes, such as phagocytosis and mechanical barriers, to implement an efficient defensive response after exposure to chemical or biological contaminants, pollutants, and contact with parasites, germs, and pathogens. Club cells (CCs) are widespread in the skin of Ostariophysi. After a predator attack or exposure to toxins and parasites, these cells can produce alarming substances. Given their effectiveness against viruses, parasites, and common skin lesions, recent studies have suggested that CCs are a component of the immune system. This study aims to immunohistochemically characterize the CCs for the first time in the skin of zebrafish, using mitogen-activated protein kinase (MAPK) p38, Toll-like receptor (TLR)2, Piscidin1, and inducible nitric oxide synthase (iNOS) peptides involved in the function of all types of vertebrate immune cells. According to our analysis, the intermediate layer of the epidermis exhibited rounded, oval, and elongated CCs, with central acidophilic cytoplasm and a spherical basophilic nucleus, that are positive to the antibodies tested. Our results may confirm that CCs could be involved in the immune function, increasing our knowledge of the immune system of teleosts.

## 1. Introduction

The immune system is represented by cellular and humoral components that defend the body from foreign substances, such as microorganisms or toxins, responding to endogenous or exogenous stimulating factors [1,2]. It is divided into an innate immune system and an adaptive immune system [3]. Innate immunity is the oldest system on the phylogenetic scale and probably originated in unicellular organisms during evolution [4]. Cellular processes, humoral components, and physical barriers, such as skin, are part of the innate immune system [5]. While the origin of innate immunity is assumed to have occurred more than 600 million years ago, some specific elements of the adaptive immune system, such as immunoglobulins (Igs) and T-cell receptors (TCRs), are relatively recent and appeared in early jawed vertebrates about 450 million years ago (Gnathostomata) [6,7,8].

Epithelia, which cover the body surfaces of vertebrates, act as a physical barrier between the interior and exterior environment. Skin encloses the body and shields it from contaminants or allergens as well as from the loss of liquids, solids, or nutrients [9]. Fish skin has a crucial role as the first line of defense against the pathogens that thrive in the aquatic environment. It is a multifunctional organ that serves as more than just a mechanical barrier, and its parts can be crucial for protection, communication, sensory perception, movement, respiration, ionic regulation, excretion, and heat regulation [9].

The fish skin consists of two layers: an outer layer, the epidermis, and an inner layer, the dermis [10]. The epidermis consists of keratinocytes and mucous cells that produce mucus and contain some antimicrobial components [11,12]. Furthermore, various specialized cells may be present, including goblet cells, sensory cells, alarm cells, and chloride cells, depending on the fish’s age, species, position on the body, the thickness of the skin, and the number of epidermal layers [13,14].

In specimens of the Ostariophysi, the epidermis is constituted of four cell types: epidermal, mucous, granular, and club cells (CCs) [15,16]. Ostariophysi is a superorder of bony fishes comprising more than 10,300 species of 1100 genera and 70 families, about 30% of all known species of Osteichthyes, 75% of all freshwater fish species, and about one-sixth of all vertebrate species. This superorder is traditionally divided into five main groups: Gonorynchiformes (dairy fishes and sandfishes; 37 species), Cypriniformes (carps and minnows; ~4262 species), Characiformes (tetra, piranha, and allies; ~2100 species), Siluriformes (catfish; ~3700 species) and Gymnotiformes (electric eel and knifefish; 225 species) [17]. Most Ostariophysi release an alarming substance from the damaged epidermis, which is produced in special epidermal cells, the CCs [18]. This is important in risk assessment and predator avoidance, and it has been considered an innovation in the successful radiation of Ostariophysi [19].

CCs are distributed throughout the epidermal layer and possess cytoplasm filled with material to be secreted and a centered nucleus [16,20]. They have been associated with several functions [21]. Zaccone et al. (1990) demonstrated the presence of serotonin (5-HT) in these cells and suggested a pheromonal function [22]. Furthermore, an antipathogenic function has been attributed to CCs [23]. The presence of chondroitin and keratin suggested a curative function in the repair of damaged tissues [21]. In addition, CCs are linked to the production, storage, and release of the alarm substance, leading to intra or interspecific alarm reactions in phylogenetically related species. The alarm reaction is triggered when individuals are injured by a predator, receiving skin wounds. This causes breakage of the CC’s cytoplasmic membrane, resulting in exposure and release of the cytoplasmic content into the water, which is detected by other individuals nearby [24]. 

The skin is involved in immune processes, acting as a mechanical and biological barrier and hosting different molecules, such as antimicrobial peptides (AMPs), neurotransmitters, and specific receptors associated with cellular damage [9,25]. This study aims to evaluate for the first time the expression of immune molecules, such as mitogen-activated protein kinase 38 (MAPK p38), Piscidin1, Toll-like receptor 2 (TLR2), and inducible nitric oxide synthetase (iNOS) in the skin CCs of Cyprinidae zebrafish (*Danio rerio,* Hamilton 1882), to highlight the possible involvement of these cells in the immune system of teleosts.

## 2. Materials and Methods

### 2.1. Samples and Tissue Preparation

Samples of zebrafish from our laboratory slide collection were processed following standard protocols for light microscopy. Two sections, 3–5 μm thick, obtained by microtome (LEICA 2065 Supercut, Nussloch, Germany, Europe), were placed on each slide. After sorting, the slides were deparaffined in xylene, and rehydrated in the descending scale of alcohols, from absolute to 30% alcohol to distilled water. 

### 2.2. Histology 

Slides were treated with a morphological stain, Mallory trichrome (04-020802 BioOptica Milano S.p.A, Milan, Italy, Europe), a histochemical stain, Alcian Blue/Periodic Acid Schiff (AB/PAS) (04-163802 BioOptica Milano S.p.A, Milan, Italy, Europe) [26], while the morphological stain Hematoxylin (H) (05-B06008/A BioOptica Milano S.p.A, Milan, Italy, Europe) was employed to counterstaining immunoperoxidase [27].

### 2.3. Immunoperoxidase

Analyses of MAPK p38, Piscidin1, and TLR2 were performed using an optical microscope and immunohistochemical techniques. Slices were exposed to anti-MAPK p38, anti-Piscidin1, and anti-TLR2 antibodies overnight in a humid environment. Slices were first washed in PBS and then incubated with a secondary antibody for 60 min. Slides were treated with diaminobenzidine (DAB) 0.02% and hydrogen peroxide 0.015% for a few minutes away from direct light. Sections were dehydrated, mounted, and evaluated using a Zeiss Axioskop 2 plus microscope (Oberkochen, Germany, Europe) and a Sony Digital Camera DSC-85 (Sony, Tokyo, Japan). As a negative control, experiments were conducted without the primary antibody.

### 2.4. Immunofluorescence and Laser Confocal Analysis

Deparaffinized and rehydrated slices were treated with bovine serum albumin (BSA) (2.5%). Then, the sections were exposed to primary antibodies against MAPK p38, TLR2, Piscidin1, 5-HT, and iNOS [28]. Subsequently, each section was assessed separately and in double-label tests. Then secondary antibodies were incubated. To prevent photobleaching, the sections were mounted with Vectashield (Vector Labs, Burlingame, CA, USA). As a negative control, experiments were run without the primary antibodies. Rat skin tissues were used as a positive control to ensure the primary antibodies’ immunopositivity [29,30].

Slices were evaluated by a confocal laser scanning microscope (Zeiss LSM DUO, Carl Zeiss MicroImaging GmbH, Jena, Germany, Europe) with a META module. Optical slices of fluorescence samples were generated by two types of lasers: helium-neon (543 nm) and argon (458 nm). The scanning rate was 62 s. The images were improved with Zen 2011 (LSM 700 Zeiss software Oberkochen, Germany, Europe). To avoid photo deterioration, each picture was snapped as rapidly as possible. To create the figure composite, a digital photo was edited using Adobe Photoshop CC ver. 2019 (Adobe Systems, San Jose, CA, USA). The “display profile” function of Zen 2011 was then used to evaluate the intensity curves of fluorescence. The information about antibodies is enclosed in Table 1.

### 2.5. Quantitative Analysis

Five slices and ten fields were evaluated from all samples of zebrafish skin to acquire data for the quantitative analysis. Observation fields were selected according to the cell’s immunopositivity, using ImageJ software ver. 1.53e. To detect the cells, the image was converted to 8 bits, a “Threshold” filter was applied to pictures, the background was eliminated, and then the number of cells was calculated using the “Analyze particles” plug-in. The number of MCs that were positive for Piscidin1, 5-HT, and TLR2 in each field was determined using SigmaPlot ver. 14.0 (Systat Software, San Jose, CA, USA). One-way ANOVA and the Student’s t-test were used to assess the normally distributed data. The data’s mean values and standard deviations, (SD) are shown: ** *p* ≤ 0.01, * *p* ≤ 0.05.

## 3. Results

**Figure 1:**
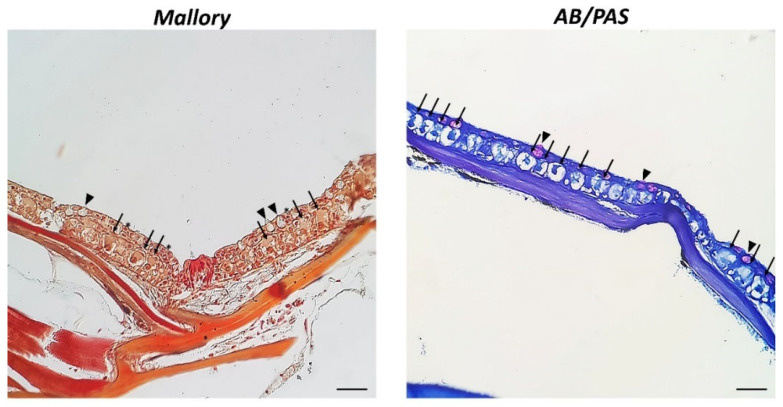
Cross sections (5 µm thick) of the zebrafish skin, 40×, scale bar 40 µm. These sections were stained with Mallory and AB/PAS. Mallory staining highlighted rounded, oval, and elongated CCs (arrows) in the intermediate layer of the epidermis with central acidophilic cytoplasm and spherical basophilic nucleus. Keratinocytes (*) are evident in the superficial stratum. The mucous cells (arrowheads) are located mainly in the superficial and medium layers, AB/PAS positive and appear purple, while CCs are AB/PAS negative, with a dark central nucleus.

Histological analysis reveals that the epidermis of zebrafish shows CCs located in the intermediate layer and appear as relatively large cells, sometimes binucleated, well organized inside the skin, with defined cellular contours, and a large nucleus centrally located, as highlighted by Mallory staining. Also, keratinocytes are evident in the surface layer. These cells present a thin contour, arranged neatly in the epidermis, and are oval, elongated, or rounded in shape. CCs do not react to AB/PAS staining, suggesting a lack of carbohydrate content (Figure 1).

**Figure 2:**
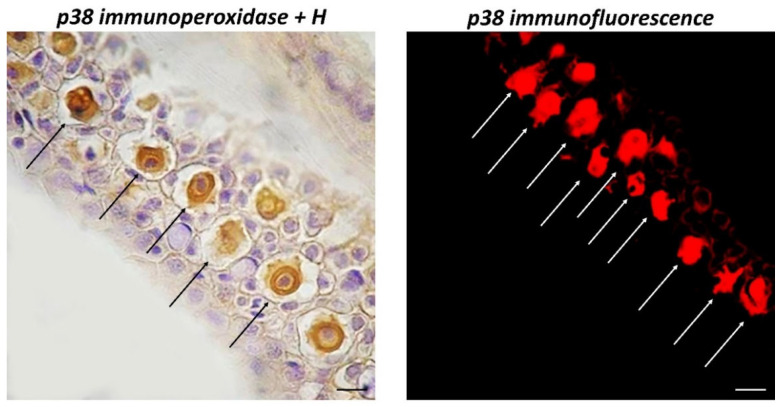
Longitudinal sections (5 μm thick) of zebrafish skin, Sections are immunohistochemically treated with MAPK p38. Immunoperoxidase counterstained by H, 100×, scale bar 100 μm. Immunofluorescence, 40×, scale bar 40 μm. Immunoreactive CCs for MAPK p38 (arrows) appear evident, with a large core centrally located. They are well organized in the intermediate epidermal layer as shown by counter colouring with H.

**Figure 3:**
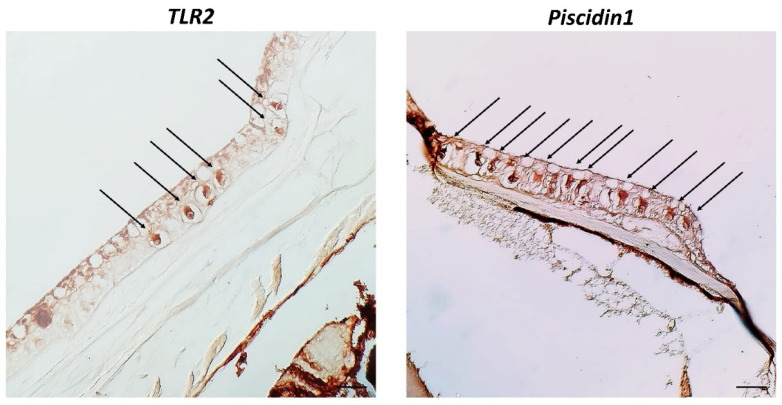
Cross sections (5 μm thick) of zebrafish skin. Sections are immunohistochemically treated with TLR2 and Piscidin1. Immunoperoxidase 40×, scale bar 40 μm. There are distinct CCs for TLR2 and Piscidin1 (arrows), with a significant core in the middle. They are neatly organized in the middle epidermal layer.

By immunoperoxidase, CCs were positive for TLR2, Piscidin1, and MAPK p38 (Figure 2 and Figure 3). Counter-staining with H of a section treated with immunoperoxidase for MAPK p38 clearly highlights immunoreactive CCs with keratinocytes and mucous cells in the outer epidermal layer in the surrounding tissue.

**Figure 4:**
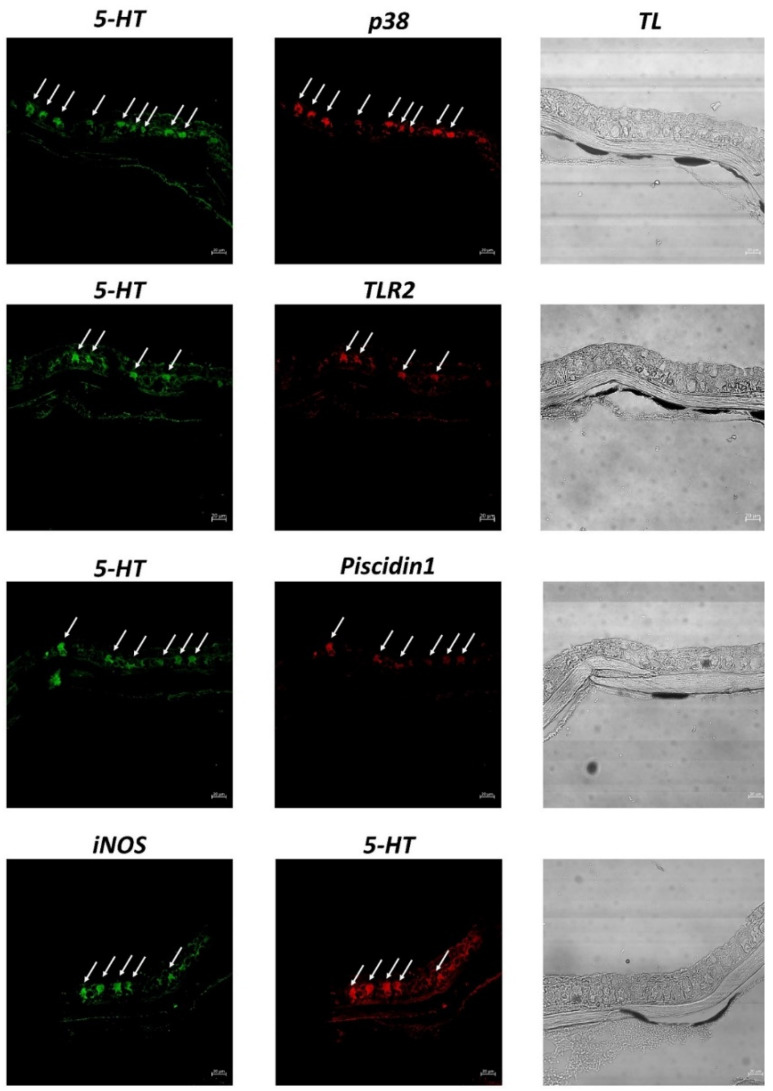
Cross sections (5 μm thick) of zebrafish skin. Sections are immunohistochemically treated with MAPK p38, TLR2, Piscidin1, 5-HT, and iNOS. Immunofluorescence 20×, scale bar 20 nm. Clear CCs immunoreactive for antibodies tested (arrows) are evident. TL = Transmitted Light.

**Figure 5:**
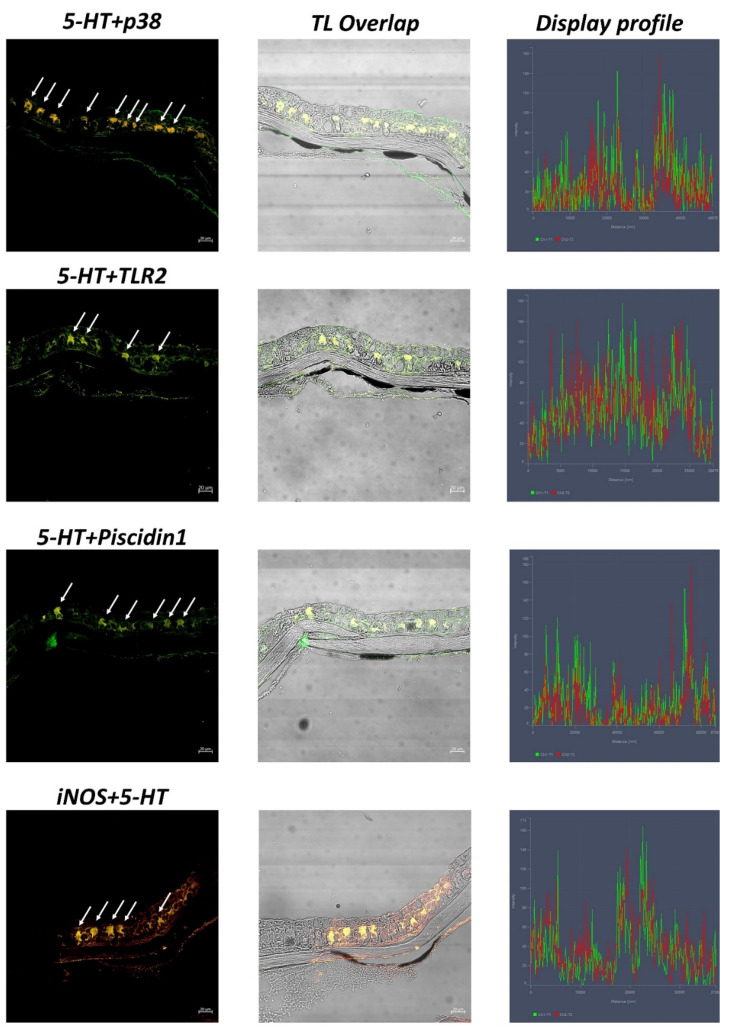
Cross section (5 μm thick) of zebrafish skin. Colocalization of antibodies tested. Immunofluorescence 20×, scale bar 20 nm. CCs immunopositive are colocalized for antibodies tested (arrows). The “display profile” function confirms these data. TL = Transmitted Light.

Under a confocal microscope, CCs appear evident and immunoreactive to the antibodies tested. Immunopositive CCs are located in the medium epidermal layer. All antibodies tested are colocalized, and thus co-expressed in the epidermal CCs of zebrafish skin, as confirmed by the display profile function, which highlighted the fluorescence peaks of antibodies (Figure 4 and Figure 5). 

Quantitative analysis revealed an equal number of positive CCs for each antibody (Table 2).

## 4. Discussion

Biological, physical, chemical, or environmental insults can cause considerable stress to aquatic organisms. Fish are particularly vulnerable to environmental changes, mainly due to overexposure through skin and gills, and are constantly in contact with the surrounding water. Fish skin presents a multilayer set of cells involved in the defense system, responding rapidly to external stimuli [31]. 

In particular, CCs, if damaged during an attack by a predator, release a substance (“alarm cue”) that causes a fear reaction in neighboring individuals [32]. A study on minnows, responding to water-soluble compounds released from damaged tissues of an injured conspecific, showed that only the injured epidermal tissue produces behavioral responses [33]. Epidermal CCs have no conduit for the release of their contents into the external environment, but they can be broken in a predator attack, releasing the alarm cue, and indicating the presence of an active predator [34]. These signals serve as a solid risk indicator and help the shoal survive encounters with predators [35]. It has been challenging to comprehend the development of CCs because it is unclear how these “signals” might be advantageous to the sender. 

Despite the fact that multiple theories have been hypothesized to explain the development of these cells, Chivers et al. (2007) provided the first substantial evidence that these cells originated as immune cells and that the alarm role may have evolved secondarily. Given their strategic structural position, epidermal CCs could serve as a first line of defense against pathogens or parasites that penetrate through the skin or promote the healing of tissues damaged by substances such as ultraviolet rays (UVR). Research has shown that exposure to pathogenic aquatic molds (*Saprolegnia ferax* and *Saprolegnia parasitica*) and parasitic larval flukes (*Uvulifer ambloplitis*) increases the density of epidermal CCs in fathead minnows (*Pimephales promelas*, Rafinesque 1820) [18,36]. 

The connection between immunity and predation is an emerging research topic. According to a recent study, fish exposed to a warning signal for four years saw an increase in the amount of lymphocytes in their blood [37]. Furthermore, the alarm signal showed antifungal properties [18]. Exposure to the warning signal, however, increases cortisol levels [38]. Khansari et al. (2018) showed that cortisol can modulate the immune response and reduce the density of CCs [39]. 

The immune function of epidermal CCs is supported by a variety of lines of evidence: (1) their strategic placement in the middle epidermal layer of the skin, which is exposed to numerous immunomodulators and environmental stressors, and serves as the first line of defense against pathogens and parasites; (2) their response to numerous immunomodulators and environmental stressors, including cortisol; and (3) the presence of numerous immunostimulants, including chondroitin and keratin sulfate, leukocytes, 5-HT, mucus, and bacteria have been observed within epidermal CCs.

Our study corroborates previous hypotheses about the immune function of CCs, showing immunoreactivity for the first time to MAPK p38, TLR2, Piscidin1, and iNOS [40,41].

The 5-HT expression, as already reported by Zaccone et al. [22], can be associated with an immune function, since this neurotransmitter is a powerful immunomodulator involved in several biological processes, such as the stimulation of mucus production by the goblet cells of mucous membranes [42]. Further modulating social responses to stressors in fish [43,44], 5-HT is involved in immune mechanisms [45], regulating the inflammatory response, recruiting immune cells, and stimulating the production of cytokines [46].

MAPKs are a class of serine/threonine kinases that play a critical role in the response to extracellular stress [47]. An essential subgroup of the MAPK family known as p38 MAPK is crucial for a variety of signaling processes, including the inflammatory response, cell differentiation, cell cycle control, and apoptosis [48,49]. It has been reported that p38 MAPK activation can be triggered by various extracellular stressors, including viral infections, environmental stress, and UV radiation [50]. Recent studies have shown the presence of p38 correlated to environmental pollutants [51] and in the ionocytes of teleosts, modulating osmoregulation [52]. Moreover, in a murine model, p38 is directly involved in inflammatory processes related to skin damage [53]. Our study is consistent with literature data, showing CCs immunopositive to p38 in zebrafish, suggesting an active role of these cells in the defense response against stressors.

Even in fish [54,55], TLRs are critical pattern recognition receptors (PRRs) that can identify a variety of pathogen associated molecular patterns (PAMPs) to activate innate immune responses against the host. TLRs are highly conserved receptors [56] that play a role in immunological response [57,58] and are present in all vertebrate classes. TLRs, in particular TLR2, have been identified in urochordates (including in the tunica and endostyle of the ascidian *Styela plicata*, Lesuer 1823) [59,60], cartilaginous fishes [61], bony fishes [62,63], and other higher vertebrates [64]. *Takifugu rubripes* (Temminck and Schlegel, 1850) and *Danio rerio* TLR profiles were compared, and a cluster of orthologous genes with considerable sequence conservation in human TLRs were discovered [65,66]. Because fish skin is the organ most exposed to stressors, epidermis cells have TLRs to mediate the immune response [58,67,68,69,70]. Our confocal microscopy investigation on *D. rerio* CCs shows the immunopositivity to TLR2. By expressing TLR2, these cells could participate in the recognition of pathogens or damage-associated antigens, thus performing an immune function.

Antimicrobial peptides (AMPs), a family of low-molecular-weight peptides and proteins, are found in nearly all life forms, from prokaryotes and eukaryotic plants to mammals [71]. In low vertebrate hosts, these peptides play a crucial role in the innate immune system [72]. Piscidins, a subgroup of amphipathic polypeptides that range in length from 18 to 46, are present in a wide range of teleosts, including the families Moronidae, Sciaenidae, Siganidae, Belontidae, Cichlidae, Percichthyidae, Latidae, Sparidae, Syngnathidae, and Latridae [73]. Fish gram-positive and gram-negative bacterial infections are effectively combated by Piscidins [74]. Fish skin has an extrinsic barrier made of a mucus layer and AMPs that acts as a barrier against surroundings that are full of pathogenic pathogens. Because these AMPs have been conserved throughout evolution and are also present in higher vertebrate skin [75]. Our study evaluates the expression of Piscidin1 in CCs of zebrafish skin. The presence of this peptide confirms previous theories on the antimicrobial power of the secretion of CCs, providing additional evidence of the possible immune function of these cells.

The smallest known bioactive molecule, nitric oxide (NO), is synthesized by nitric oxide synthase (NOS) and can be produced by several cell types. NO is crucial for controlling immunological activity, host defense, vascular function, and neurotransmission [76]. Neuronal nitric oxide synthase (nNOS), inducible nitric oxide synthase (iNOS), and endothelial nitric oxide synthase (eNOS) are the three NOS isoforms that have been identified [77]. Both nNOS and eNOS are calcium-dependent enzymes that are mostly expressed in neurons and epithelial cells, respectively. Contrarily, calcium-independent iNOS can be released upon cytokine or other stimuli-induced activation. NO is a significant proinflammatory mediator with immune system effects, being involved in the immunoinflammatory process [77]. The primary effector cells implicated in the antimicrobial effects of NO are macrophages, and also neutrophils, monocytes, and epithelial cells [78]. Studies on Ostariophysi suggested that purine N-oxides act as chemical alarm signals and that the functional group of nitric oxide acts as the main molecular trigger. One study found that the exposure of *Ictalurus punctatus* (Rafinesque, 1818) to a compound of hypoxanthine-3-N-oxide resulted in significant increases in species-specific antipredator behavior. In addition, two nonostariophysan species known to have chemical alert signals did not show any increase in antipredator behavior in response to hypoxanthine-3-N-oxide [35,79]. In our study, we find positively marked epidermal CCs in zebrafish with iNOS, in accordance with their role in the warning signals typical of CCs and suggesting their involvement in immune responses of Ostariophysi.

The accumulation of evidence indicates that CCs may be innate immune cells involved in different immune functions. Our study evaluates for the first time the immunohistochemical expression by confocal microscopy of different immune molecules, suggesting a role is played by CCs in the immune system of Ostariophysi. Furthermore, the colocalization of the antibodies tested, confirmed by the display profile function of the confocal microscope, corroborates our results. These findings can provide further information about these peculiar cells and deepens the knowledge about the immune system of teleosts. The observed immunopositivity of these cells to the antibodies tested confirms the presence of the receptors on the cell surface, and, since these receptors play a crucial role in immunity, their expression might suggest an effective involvement of CCs in the Ostariophysi defensive response. However, additional molecular biology, genetics, and in vivo studies are needed to further validate our data.

## Figures and Tables

**Table 1:** Antibodies data.

Antibody	Supplier	Dilution	Animal Source
MAPK p38	Sigma-Aldrich, Inc., St. Louis, MO, USA	1:100	Rabbit
Piscidin1	GenScript Biotech Corporation, Rijswijk, Netherlands, Europe. Produced on demand	1:50	Rabbit
TLR2	Active Motif, La Hulpe, Belgium, Europe	1:125	Rabbit
5-HT	Santa Cruz Biotechnology, Inc., Dallas, TX, USA	1:50	Mouse
5-HT	Sigma-Aldrich, Inc., St. Louis, MO, USA	1:300	Rabbit
iNOS	Santa Cruz Biotechnology, Inc., Dallas, TX, USA	1:200	Mouse
Goat anti-Rabbit IgG Peroxidase conjugated	Sigma Aldrich, Saint Louis, MO, USA	1:100	Goat
Alexa Fluor 488 Donkey anti-Mouse IgG FITC conjugated	Molecular Probes, Invitrogen	1:300	Donkey
Alexa Fluor 594 Donkey anti-Rabbit IgG TRITC conjugated	Molecular Probes, Invitrogen	1:300	Donkey

**Table 2:** Quantitative analysis results (mean values ± standard deviations; *n* = 3).

		No. of CCs ^1^
Immunoperoxidase	MAPK p38	2769.93 ± 345.98 *
	TLR2	2819.05 ± 289.56 **
	Piscidin1	2681.94 ± 3 05.67 *
Immunofluorescence	MAPK p38	3117.54 ± 319.29 *
	TLR2	3123.55 ± 279.06 *
	Piscidin1	3114.81 ± 385.90 **
	5-HT	3185.46 ± 322.73 **
	iNOS	3067.84 ± 341.92 *
Colocalization	5-HT+MAPK p38	3067.84 ± 341.92 *
	5-HT+TLR2	3067.84 ± 341.92 *
	5-HT+Piscidin1	3067.84 ± 341.92 *
	5-HT+iNOS	3067.84 ± 341.92 *

** *p* ≤ 0.01; * *p* ≤ 0.05; ^1^ Comparison of the means was carried out by applying One-way ANOVA and Student’s t-test.

## Data Availability

Not applicable.
